# Plasma S-Adenosylmethionine Is Associated with Lung Injury in COVID-19

**DOI:** 10.1155/2021/7686374

**Published:** 2021-12-16

**Authors:** Evgeny Vladimirovich Kryukov, Alexander Vladimirovich Ivanov, Vladimir Olegovich Karpov, Valery Vasil'evich Aleksandrin, Alexander Mikhaylovich Dygai, Maria Petrovna Kruglova, Gennady Ivanovich Kostiuchenko, Sergei Petrovich Kazakov, Aslan Amirkhanovich Kubatiev

**Affiliations:** ^1^Burdenko Main Military Clinical Hospital, Ministry of Defense, Gospitalnaya Sq., 3, Moscow, 105229, Russia; ^2^Institute of General Pathology and Pathophysiology, Baltiyskaya St., 8, Moscow, 125315, Russia; ^3^Sechenov First Moscow State Medical University, Trubetskaya St., 8-2, Moscow, 119991, Russia; ^4^Regional Clinical Hospital, Lyapidevsky St., 1, Barnaul, 656024, Russia

## Abstract

**Objective:**

S-Adenosylmethionine (SAM) and S-adenosylhomocysteine (SAH) are indicators of global transmethylation and may play an important role as markers of severity of COVID-19.

**Methods:**

The levels of plasma SAM and SAH were determined in patients admitted with COVID-19 (*n* = 56, mean age = 61). Lung injury was identified by computed tomography (CT) in accordance with the CT0-4 classification.

**Results:**

SAM was found to be a potential marker of lung damage risk in COVID-19 patients (SAM > 80 nM; CT3,4 vs. CT 0-2: relative ratio (RR) was 3.0; *p* = 0.0029). SAM/SAH > 6.0 was also found to be a marker of lung injury (CT2-4 vs. CT0,1: RR = 3.47, *p* = 0.0004). There was a negative association between SAM and glutathione level (*ρ* = −0.343, *p* = 0.011). Interleukin-6 (IL-6) levels were associated with SAM (*ρ* = 0.44, *p* = 0.01) and SAH (*ρ* = 0.534, *p* = 0.001) levels.

**Conclusions:**

A high SAM level and high methylation index are associated with the risk of lung injury in patients with COVID-19. The association of SAM with IL-6 and glutathione indicates an important role of transmethylation in the development of cytokine imbalance and oxidative stress in patients with COVID-19.

## 1. Introduction

Predictive factors of and markers for the severity of new coronavirus infection (COVID-19) are being actively studied. Large-scale studies have revealed the associations of several factors (age, heart failure, chronic obstructive lung disease, cancer, obesity, chronic kidney disease, diabetes mellitus, stroke, and comorbidities) with an unfavorable course of this disease [1–4]. Endothelial dysfunction (ED) plays an important role in the pathogenesis of various vascular diseases and COVID-19 [5, 6]. Therefore, increased attention is being paid to the role of endothelial, vascular (primarily associated with coagulation), and inflammatory biomarkers, including circulating vascular cell adhesion molecule-1 (sVCAM-1), Von Willebrand factor (vWF), heparan sulfate (a product of endothelial surface glycocalyx degradation), and P-selectin [7, 8]. Furthermore, studies have focused on coagulation, acute phase, and inflammatory biomarkers widely used in clinical practice, such as D-dimer, platelet count (PLT), C-reactive protein (CRP), interleukin 6 (IL-6), and ferritin [9]. S-adenosylmethionine (SAM) and S-adenosylhomocysteine (SAH) influence vital processes including regulation of the expression of cytokine and inflammatory protein genes and proliferation of viral particles. SAM is a methyl group donor in all transmethylation reactions and is required for the synthesis of polyamines. SAH and a product of its hydrolysis, homocysteine (Hcy), are biological inhibitors of transmethylation. Therefore, the SAM/SAH ratio is known as the methylation index. Notably, an increase in the SAH level and a decrease in the methylation index are considered markers of ED in both experimental models and individuals with chronic cardiovascular diseases [10–13].

In severe clinical cases, several manifestations of COVID-19 are similar to those of sepsis. An increase in the plasma SAM level has been observed in a rat septic shock model [14]. Furthermore, in a previous study, patients with sepsis presented significantly higher plasma SAM and SAH levels than control participants and sepsis nonsurvivors presented significantly higher plasma SAM and SAH levels than survivors [15].

A recent report suggested the role of high Hcy levels as a risk factor for severity or complications in COVID-19 [16, 17]. Another study showed a significant correlation between Hcy levels and imaging progression on chest computed tomography (CT) from COVID-19 patients [18]. In addition, the use of a complex of B vitamins led to a decrease in the level of Hcy in COVID-19 patients; this was associated with a decrease in the period of fever and normalization of the level of D-dimer and C-reactive protein (CRP) [19]. As SAM and SAH are precursors of Hcy, the above findings suggest that these metabolites may also be considered markers of or severity factors of COVID-19.

In addition, there are a number of arguments indicating the important role of SAM and SAH in acute respiratory syndrome coronavirus-2 (SARS-CoV-2). Its nonstructural proteins (nsp) 14 and 16, i.e., (guanine-N7)-methyltransferase (N7-Mtase) and 2′-O-methyltransferase (2′-OMTase), respectively, are SAM-binding proteins; they play a crucial role in viral transmission and viral replication [20, 21]. 2′-O methylation prevents virus detection by cell innate immunity mechanisms and viral translation inhibition [22]. It has also been suggested that SAM/SAH (methylation index) balance is a regulator of 2′-OMTase activity and raises the possibility that SAHH inhibitors might interfere with coronavirus replication cycle [22]. Synthetic inhibitors of N7-Mtase and 2′-OMTase are considered as promising antiviral drugs [23–25]. It was also proposed to use the restriction of the bioavailability of methionine as the main substrate for the synthesis of SAM by treating a COVID-19 patient with oral recombinant methioninase [26].

According to a recently proposed hypothesis, SARS-CoV-2 induces changes in host's one-carbon metabolism and methyl-group availability. Disruption of transmethylation by SARS-CoV-2 will lead to a decrease in intracellular SAM concentration. This limits the ability of cells to synthesize glutathione (GSH), a key intracellular antioxidant [27]. Recently, a low GSH level has been reported to be a marker for the risk of severe lung injury associated with COVID-19. [28].

To the best of our knowledge, there are on clinical studies of SAM and SAH levels in patients with COVID-19. In the present study, we aimed to investigate the possibility of an association of the plasma levels of SAM and SAH with the severity of lung injury, using some routinely used biomarkers such as D-dimer, C-reactive protein (CRP), interleukin-6 (IL-6), and ferritin and aminothiols, including Hcy and GSH in patients with COVID-19 at admission.

## 2. Methods

### 2.1. Patients

This study included 56 COVID-19 patients who were admitted in the pulmonary department of the Burdenko Main Military Clinical Hospital from September 2020 to December 2020. The study was approved by the local institutional ethics committee. Informed written consent was obtained from each patient. The reporting of this study conforms to STROBE guidelines [29].

The patients were diagnosed according to the World Health Organization's interim guidelines for COVID-19. The main inclusion criterion was a confirmed primary coronavirus infection. Exclusion criteria included exacerbated cardiovascular disease, HIV infection, hepatitis B and C, terminal cancer, and decompensated renal failure. All patients undergoing treatment were discharged after recovery from the infection and improvement in their general condition. On admission, the patients were divided into mild, moderate, and severe condition groups according to their complaints and the results of the initial examination. On admission, the patients were prescribed a standard therapy in accordance with the recommendations of the Ministry of Health of the Russian Federation. The therapy included steroids (dexamethasone intravenous 8, 16, or 32 mg; prednisolone 30 or 40 mg tablets; and intravenous 500 or 1000 mg), anticoagulants (enoxaparin subcutaneous 0.4 or 0.8 mg, trombovazim 2 × 800 or 2 × 1600 U), paracetamol (0.5 g, for fever >38°C), gastroprotectant (omepaminsrazol 20 or 40 mg), and vitamins Angiovit 1–2 tablets (1 tablet: B_9_ 5 mg; B_6_ 4 mg; B_12_ 0.006 mg) and recommended 4–5 h prone position and oxygen support.

Chest CT were performed at 48 h after the administration of the patients using the Optima CT660 tomograph (GE Healthcare, USA), from the level of the thoracic entrance to the level of the diaphragm, and completed at the end of inspiration. The scanning parameters were as follows: tube voltage, 120 kV; tube current, 114–350 mA; layer thickness, 5 mm. At the end of scanning, a thin layer image with a layer thickness of 2.5 mm was automatically reconstructed and recorded as DICOM image data. The reconstruction algorithm used was with a field of view of 360 mm × 360 mm and a matrix of 512 × 512. Image browsing and multiplane reconstruction were performed using GE AW VolumeShare software v.4.6; images of the lungs (window width, 1500; window level, 500) and the mediastinum (window width, 350; window level, 35–40) were also observed using the same software. Image analysis was performed following the standard protocol described elsewhere [30]. The degree of lung damage was then assessed using the following scoring system based on the percentage of lobar involvement: <5% (CT0), 5%–25% (CT1), 26%–49% (CT2), 50%–75% (CT3) and >75% (CT4) [31]. Based on the data of an objective study of the respiratory function and blood oxygen saturation, patients were categorized into mild, moderate, and severe groups [32].

### 2.2. Laboratory Procedures

On admission, venous blood was collected in 0.105 M sodium citrate tubes and centrifuged at 3000 g for 15 min. Following that, 1.45 ml of plasma was mixed with 0.05 ml of 3 M acetic acid, and the samples were frozen at -80°C and stored until analysis.

All patients were confirmed COVID-19 positive by using SARS-CoV-2 nucleic acid detection kit “AmpliPrime® SARS-CoV-2 DUO” (Next Bio, Russia) and PCR analyzer Rotor-Gene Q (QIAGEN, Germany).

GSH and Hcy levels were determined by liquid chromatography as described in a previous study [33]. SAM and SAH levels were determined by liquid chromatography–fluorescence detection as described in a previous study, with some modifications [34]. A UPLC ACQUITY system (Waters, Milford, MA, USA) was used in both these cases. Zorbax Eclipse plus C18 Rapid Resolution HD column (150 mm × 3 mm, 1.8 *μ*m; Agilent, Santa Clara, USA) was used for chromatography. Flow rate was 0.37 ml/min at a temperature of 35°C. Mobile phases were 40 mM acetic acid with 5 mM KH_2_PO_4_+ 25 *μ*M heptafluorobutyric acid and acetonitrile. Chromatography was performed using a linear acetonitrile gradient (2%–15%) for 5 min. The column was regenerated with 50% acetonitrile for 1.5 min and equilibrated with 2% acetonitrile for 6.5 min.

Data collection and primary processing (identification and integration of the chromatographic peaks) were performed in MassLynx v4.1 (Waters, USA). Statistical data analysis was performed using SPSS Statistics v. 22 (IBM, USA). Data on age and clinical and biochemical indicators are expressed as medians [1st; 3rd quartile]. Differences in the levels of these parameters between the patient groups were determined using rank Mann-Whitney *U* and Kruskal-Wallis tests. Spearman correlation coefficient (*ρ*) was used to describe the association between different variables. Comparison of binomial indicators (variable analysis) was carried out via relative risk (RR) and odds ratio (OR); *p* < 0.05 was considered to indicate a significant difference.

## 3. Results

The median patient age was 61 [51; 67.5] years, with an age range of 20–88 years. Among the patients, 20 were active military personnel, 17 were retired military personnel (working), and the remaining 19 were retired. There were no smokers or regular consumers of drugs among the patients. Most of those admitted (*n* = 34, 61%) had a mild infection, 34% (*n* = 19) were admitted with moderate to severe infection, and 5% (*n* = 3) with severe infection; therefore, the last two groups were subsequently merged. The incidence of chronic cardiovascular diseases and heart failure was high (29% and 23%, respectively) in the entire cohort, but there were no significant differences in the incidence of these and other anamnestic factors by age and sex distribution between the groups (Table 1). Furthermore, there were no significant differences in the SAM, SAH, and Hcy levels and SAM/SAH. However, the level of GSH in the mild group was higher than that in the moderate-to-severe/severe group; consequently, the SAM/GSH was higher in the latter group. Moreover, patients with moderate-to-severe/severe COVID-19 presented higher hematocrit (HCT) and leukocyte index (LI) than those with mild COVID-19.

The general characteristics of the patients grouped based on CT findings are presented in Table 2. The majority (77%) of the patients were men, and all were men in the CT3,4 group. The degree of lung damage corresponded to CT4 only in two patients. Therefore, the groups CT3 and CT4 were subsequently merged. On admission, two patients underwent resuscitation/intensive therapy. A significant proportion of patients were previously diagnosed with arterial hypertension (24 out of 56, or 43%) and atherosclerosis (16 out of 56, or 29%). Significant differences were found between these groups in a number of laboratory indicators. In group CT3,4, there was a significant increase in erythrocyte sedimentation rate (ESR) and the levels of aspartate aminotransferase (AST), alanine aminotransferase (ALT), IL-6, CRP, SAM, and SAM/GSH. Increased levels of SAM/SAM and SAM/GSH were observed in the CT2 group.

**Figure 1:**
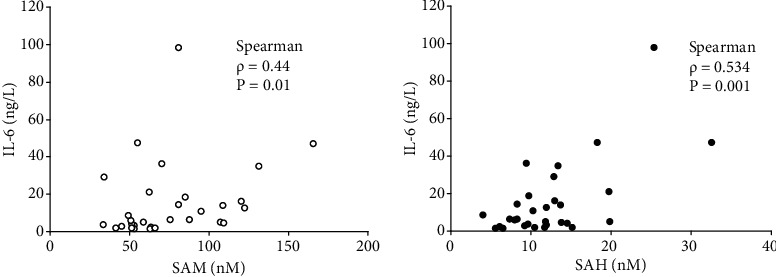
Association of SAM and SAH with IL-6 levels in COVID-19 patients.

In the patient cohort, numerous associations among laboratory parameters were identified. The only indicator that had a clear association with age was ferritin (*ρ* = 0.619, *p* = 0.004). In addition, its level had a significant positive correlation with ALT (*ρ* = 0.644, *p* = 0.007) and AST (*ρ* = 0.684, *p* = 0.003) levels. SAM and creatinine levels were also significantly associated with each other (*ρ* = 0.454, *p* = 0.00045). Spearman rank correlation revealed a positive association of SAM (*ρ* = 0.44, *p* = 0.01) and SAH (*ρ* = 0.534, *p* = 0.001) with IL-6 levels (Figure 1). No significant association between SAM and SAH was observed (*ρ* = 0.217, *p* = 0.108). Further, there was no significant influence of sex and age on SAM, SAH, and IL-6 levels.

**Figure 2:**
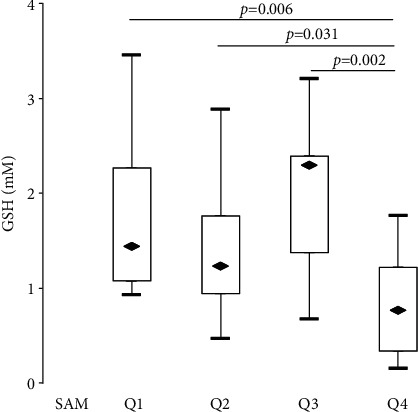
Distribution of GSH level at quartilization of patients with SAM levels (Q1: 24.3–51.3 nM, Q2: 51.4–62.8 nM, Q3: 63.2–37.9 nM, and Q4: 95.3–301.2 nM).

We did not find an association between SAM and GSH in the entire cohort of patients; however, after excluding two patients with abnormally high GSH levels (6.9 and 9.9 *μ*M), a negative association of these analytes was found (*ρ* = −0.343, *p* = 0.011). Furthermore, after quartilizing the cohort of patients by the SAM level, it was found that among the patients in the highest quartile, the GSH level was lower than in the other groups (0.78 vs. 1.22–2.23 *μ*M, Figure 2). Thus, it was shown that for a high level of SAM (>95 nM), a decrease in the level of plasma GSH is characteristic.

**Figure 3:**
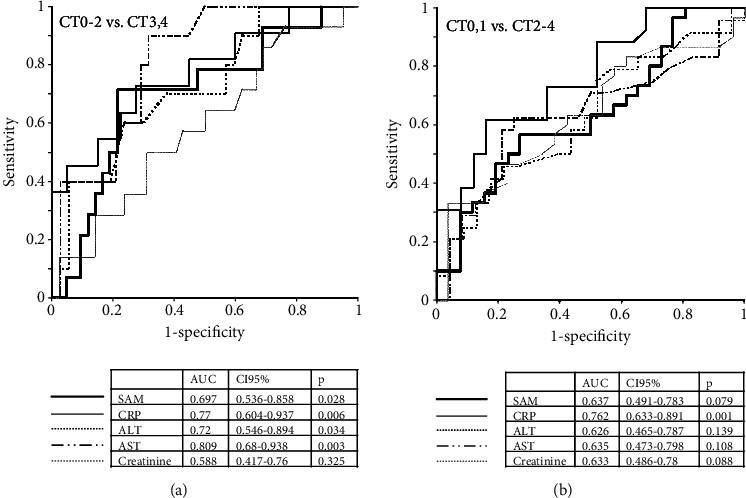
ROC analysis of the laboratory variables to compare their diagnostic performance in detecting lung injury in COVID-19. (a) CT0-2 vs. CT3,4; (b) CT0,1 vs. CT2-4.

When the cohort of patients was divided into two groups (CT0-2 and CT3.4), the ROC analysis revealed a fairly good SAM classification ability (AUC, 0.697; sensitivity, -0.714; specificity, - 0.786 at cut-off 78.1 nM), although it was inferior to other markers (CRP, ALT, and AST) (Figure 3(a)). When the CT0,1 group was compared with CT2-4, the SAM, ALT, and AST levels were not sensitive enough markers (see Figure 3(b)). At the same time, the SAM/SAH ratio (AUC: 0.659, CI95%: 0.515-0.803, *p* = 0.042; sensitivity –0.567, specificity –0.808 at cut-off 5.88) and, especially, SAM/GSH (AUC: 0.719, CI95%: 0.584–0.854; *p* = 0.005, sensitivity –0.767, specificity –0.615 at cut-off 36.3 nM/*μ*M) demonstrated relatively satisfactory performance of the ROC analysis.

To determine the effectiveness of SAM as a marker for the risk of lung injury, we calculated the RR and OR by varying the threshold values for indicators such as the SAM level, SAM/SAH, and SAM/GSH. These results are presented in Table 3. Most of the patients (64%) with severe lung damage (CT3,4) had SAM > 80 nM, and only 21% of the patients with CT0-2 had SAM > 80 nM. The majority of patients (67%) in the CT3.4 group had a high methylation index (SAM/SAH > 6); whereas, in the CT0-2 group, there were only 19%. Furthermore, a high SAM/GSH (>60 nM/*μ*M) was observed more often in the CT3,4 group than in the CT0-2 group (50% and 19%, respectively). Thus, an elevated SAM level and SAM/SAH and SAM/GSH ratios have been associated with an increased risk of severe lung injury (CT3,4).

## 4. Discussion

The key results of our study are as follows. (1) An increased level of SAM or SAM/SAH ratio is associated with the risk of severe lung injury in COVID-19. (2) There is a negative association between the level of SAM and that of GSH. (3) SAM and SAH are correlated with the IL-6 level. The first finding is consistent with the results of a metabolomic study of blood plasma in patients with COVID-19 [35], which showed an increase in the SAM level in critically ill patients (i.e., those in the ICU) compared with that in the mild, moderate, and control groups. These results are broadly consistent with previous studies of experimental endotoxinemia-induced septic shock, which showed a significant increase in plasma and liver SAM levels [14, 36]. The authors suggested that this effect is due to the inhibition of transmethylases due to predominance of catabolic over anabolic processes [14]. This explains the lack of significant changes in the SAH level in this model. This is confirmed by the fact that in addition to an increase in the expression level of methionine adenosyltransferase (an enzyme that synthesizes SAM), endotoxienmia caused a significant decrease in the expression of glycine N-methyltransferase, which is the most active liver methyltransferase [36]. It is also unlikely that increase in the SAM level was due to inhibition of plasma pool utilization by the kidneys, as there was no increase in the SAH level, which is also mainly utilized through the kidneys [37]. In addition, an increase in the SAM levels can be caused by increased exocytosis of this metabolite during cellular damage.

SAM is an allosteric activator of the Hcy to cysteine pathway, which is required for GSH synthesis. So, an in vitro model of lipopolysaccharide-activated monocytes showed an increase in SAM levels on the first day, accompanied by an increase in GSH levels [38]. In addition, it was demonstrated that the addition of SAM to macrophage culture attenuated the decrease in GSH levels and the expression of GSH-synthesizing enzymes, caused by the presence of lipopolysaccharide (LPS) [39].

Another important aspect of SAM action is that it inhibits the activation of gamma-glutamyl transferase (GGT), an enzyme that hydrolyzes GSH. SAM intake prevents GSH degradation caused by GGT activation in experimental models of cholestasis-induced sepsis and with the administration of the toxin cyclosporin A [40, 41]. This effect has been confirmed in clinical studies of patients with cholestasis and chronic kidney disease, wherein the intake of SAM was accompanied by a decrease in the serum GGT level [42]. Besides GGT, SAM has a positive effect on the GSH cell pool by increasing in the gamma-glutamylcysteine ligase (GGL) levels [43]. GGL is a key enzyme in GSH synthesis.

All this seemingly contradicts the negative association between the SAM and GSH levels revealed in this study. In contrast, in our study, an increase in the SAM/GSH ratio was found to be associated with an increased risk of lung damage, and patients with more than 50% of lung damage were characterized by both increase in the SAM level and decrease in the GSH level. Thus, in the model of endotoxinemia, a decrease in the GGL activity in the liver and GSH level were observed [44], despite the increase in the SAM level as noted above [14]. An increase in the SAM level may reflect disturbances in the methylation processes, including epigenetic mechanisms regulating the expression of genes related to GSH metabolism, which may be opposite to the direct protective effect of SAM. Although the details of the regulation of GGT expression have not been extensively studied, an association of the level of this enzyme with DNA methylation of a number of genes has been revealed [45]. Recent studies have shown that in SARS-CoV-2-positive patients, there is no decrease, but, on the contrary, an increase in the level of GGT. Moreover, the level of GGT in patients with severe pneumonia was significantly higher than in patients with mild pneumonia [46, 47]. This explains the decrease in the GSH levels and may indicate that the increase in the level of SAM does not play a significant protective role in COVID-19.

It can also be assumed that DNA hypomethylation plays a role in reducing the GSH level by inhibiting GGL expression, as these effects have been previously shown in liver cell culture, accompanied by an increase in the SAM level (under the action of the carcinogen 3-methylcholanthrene) [48]. Thus, the negative association between SAM and GSH, most likely, is a reflection of the important role of transmethylation in GSH metabolism. Further studies should focus on the mechanisms of epigenetic regulation of GGT and GGL expression, as well as the activity of methyltransferases in COVID-19.

Notably, a positive association of IL-6 with SAM and SAH was also found. IL-6 can exhibit both proinflammatory and anti-inflammatory properties, but an increase in its level in COVID-19 primarily plays a proinflammatory role, since it is an active participant in the so-called “cytokine storm” [49, 50]. Numerous clinical studies show an association of elevated IL-6 levels with the severity of COVID-19, which is consistent with our results [51]. The association of IL-6 levels with the severity of lung damage in both COVID-19 and other pneumonias has been shown in previous studies [52, 53]. GSH, in turn, inhibited IL-6 expression in LPS-activated alveolar macrophages [54].

SAM has a significant effect on the expression of IL-6, but the results of different studies are ambiguous. It was previously shown that SAM increases IL-6 production and GSH synthesis in an LPS-activated monocyte culture, but this effect is blocked by the inhibition of SAH hydrolase, an enzyme that cleaves SAH to Hcy and adenosine (Ado), or by the inhibition of methionine adenosyltransferase [55–57]. Both the above mentioned studies showed that the effect of SAM was suppressed by the inhibition of the adenosine A2 receptor. These studies concluded that the stimulation of IL-6 expression was due to an increase in the level of Ado and signaling from the A2 receptor. Ado directly caused an increase in IL-6 production in activated monocytes [56]. However, we do not yet have data on whether the increase in SAM levels is accompanied by an increase in the levels of Ado in COVID-19 patients. Indirectly, an even closer association of the levels of SAH (the precursor of Ado) with IL-6 indicates this possibility.

However, other studies on LPS-activated macrophage culture have shown that SAM significantly inhibits IL-6 expression [58, 59]. This process involves the inhibition of mitogen-activated protein kinases (MAPK: ERK1/2, JNK1/2, p38) and is accompanied by an increase in global DNA methylation [58]. In addition, nonspecific inhibition of DNA transmethylases suppressed this effect of SAM. Although these results may explain the association of SAM levels with IL-6 in COVID-19 patients, the association of SAH with IL-6 remains unclear, since SAH is a transmethylase inhibitor that should cause a global decrease in DNA methylation.

Due to the close metabolic relationship, plasma SAM and SAH levels show a fairly high correlation in normal conditions [10, 34, 60]. However, since our study showed that COVID-19 patients with severe lung damage showed an increase in plasma SAM levels, but no significant increase in SAH levels, this clearly indicates a dysregulation of transmethylation in COVID-19.

The association of SAM levels with plasma creatinine is not surprising, since the formation of the latter requires the participation of SAM as a methyl group donor. It was previously shown that serum creatinine levels at baseline were higher in patients requiring ICU admission and mechanical ventilation, and therefore, this indicator found as independent risk factor for in-hospital death too [61]. Although in our work the diagnostic value of creatinine was not revealed, further study of SAM as a factor in creatinine metabolism may be of interest.

In our study, when patient groups by the severity of disease were compared, among all laboratory parameters, only the increase in LI (neutrophils/(monocytes + lymphocytes)) was found to be significant in patients with moderate-to-severe/severe COVID-19. This, in principle, is consistent with the results of a large study, which reported that an increase in the neutrophil/lymphocyte ratio is a prognostically unfavorable marker for COVID-19 outcome [3]. The underlying mechanisms is not yet clear, but it is considered that inappropriate neutrophil extracellular trap (NET) production plays a key role [62]. The role of GSH in NETs is not yet clear. Glutathionylation of actin, tubulin, and possibly other proteins has been shown to inhibit polymerization and NET formation, as shown in vitro in the presence of glutaredoxin 1 and under GSH-reductase enzyme deficiency [63, 64]. In addition, an increase in the intracellular level of SAM through an increase in the formation of polyamines [65, 66] can have a potentiating effect on NET formation and stabilization.

This study had some limitations. It should be noted that the levels of SAM and SAH presented herein are only approximate values, as these metabolites are rather labile and the method of sample preparation and analysis influence the results. The small number of patients, the heterogeneity of patient groups, and the lack of follow-up of patients limit the generalizability of the findings in a single-center study. Our findings indicate the importance of assessing the SAM level and SAM/SAH as markers of COVID-19 prognosis or the use of methyltransferase inhibitors for the treatment of COVID-19.

## 5. Conclusion

Since the methylation (capping) of viral RNA is necessary for its life cycle, the role of this metabolite in the pathophysiology of COVID-19 is not entirely clear. Elevated SAM level can be considered as a marker for the risk of lung damage in patients with COVID-19 and, most likely, a factor associated with the development of the inflammatory process and with a decrease in the main cellular antioxidant GSH. On the other hand, there are several reasons to consider an increase in SAM levels as an anti-inflammatory response of the body. The association of SAM and SAH with IL-6 suggests that they play an important role in transmethylation toward the development of cytokine imbalance in COVID-19, but more research is needed to identify the pathogenetic and therapeutic potential for correcting SAM levels.

## Figures and Tables

**Table 1:** Comparative characteristics of patients with different severities of coronavirus infection.

	Mild	Moderate+severe	*p*
*N*	34	22	
Age, y	62 [50.8; 67.8]	57.5 [50.5; 64.0]	0.775
Age > 69 y, *N*	7	5	
Sex, man (%)	24 (71)	19 (86)	0.171
Lung CT:			
0,1	19	7	0.078
2	10	6	0.865
3	5	7	0.129
4	0	2	0.073
Alcohol consumption	3	1	0.545
Chronic cardiovascular diseases	9	7	0.667
Heart failure	8	5	0.944
Diabetes mellitus	3	1	0.549
Cancer	5	2	0.535
Chronic kidney disease	3	0	0.153
Chronic obstructive lung disease	2	0	0.246
Comorbidities (3 and more)	8	4	0.631
*HCT, %*	*41 [33; 44]*	*43 [42; 45]*	*0.011*
*LI*	*2.15 [1.53; 3.3]*	*4.05 [2.55; 5.7]*	*0.005*
*GSH, μM*	*1.74 [1.07; 2.32]*	*0.99 [0.68; 1.40]*	*0.011*
Hcy, *μ*M	7.9 [6.3; 9.3]	7.3 [6.0; 11.9]	0.987
SAM, nM	61 [50; 79]	75 [52; 115]	0.164
SAH, nM	13.6 [10.5; 21.6]	14.0 [9.9; 19.5]	0.973
SAM/SAH	4.5 [3.0; 7.2]	6.0 [3.6; 8.6]	0.196
*SAM/GSH, nM/μM*	*37.8 [28.8; 63.2]*	*60.6 [41.7; 254]*	*0.016*

CT: computed tomography; GSH: glutathione; HCT: hematocrit; Hcy: homocysteine; LI: leukocyte index; SAH: S-adenosylhomocysteine; SAM: S-adenosylmethionine.

**Table 2:** Comparative characteristics of patients with different degrees of lung damage on admission.

	CT0, 1	CT2	CT3,4	*p* ^Kruskal-Wallis^
*N*	26	16	14	
Age, y	64.5 [51.3; 71.8]	60.5 [52.8; 67.5]	56.0 [49.3; 62.8]	
Sex, man (%)	18 (72%)	11 (69%)	14 (100%)^‡^	
Arterial hypertension (%)	13 (23%)	6 (11%)	5 (9%)	
Diabetes mellitus (%)	3 (12%)	2 (11%)	2 (13%)	
Atherosclerosis (%)	9 (36%)	3 (16%)	4 (29%)	
SpO_2_, %	97 [95; 98]	96 [94; 97.3]	97 [96; 98]	
HGB, g/l	140 [128; 161]	130 [119; 161]	147 [144; 149]	
HCT, %	42 [37; 45]	43 [36; 47]	43 [42; 43]	
RBC, 10^12^/l	5.0 [4.3; 5.2]	5.0 [3.6; 5.4]	4.9 [4.5; 5.1]	
PLT, 10^9^/l	261 [216; 326]	237 [163; 275]	253 [210; 304]	
MCV, fl	85.5 [82.5; 88.0]	87 [85; 90]	86.5 [84; 91.1]	
MCH, pg/cell	30 [28.3; 30.2]	30.5 [28.2; 31.7]	29.6 [29.0; 30.0]	
MCHC, g/l	348 [335; 360]	343 [329; 362]	347 [335; 354]	
WBC, 10^9^/l	6.14 [4.68; 8.88]	5.2 [3.7; 5.9]	7.7 [4.4; 10.9]	
LI	2.4 [1.8; 3.4]	2.9 [1.7; 4.5]	3.6 [3.2; 5.7]	
ESR, mm/h	34 [19; 52]	44 [18; 71]	82 [76; 86] ^‡£^	
ALT, U/l	28 [20; 37.5] *n* = 23	30.5 [21.5; 54.5] *n* = 14	47.5 [29.3; 142.3]^‡^ *n* = 10	
AST, U/l	29.5 [26.8; 35.4] *n* = 24	29.0 [24.5; 53.3] *n* = 14	50.5 [37.8; 101.5]^‡£^ *n* = 10	0.012
D-dimer, mg/l	0.93 [0.51; 1.53] *n* = 6	0.71 [0.46; 0.82] *n* = 7	1.18 [0.72; 1.69] *n* = 7	
CRP, mg/l	7.4 [1.8; 24.0] *n* = 25	32.0 [6.1; 64.8]^‡^ *n* = 15	71.8 [27.0; 118.5]^‡^ *n* = 11	0.003
IL-6, ng/l	4.85 [3.00; 18.5] *n* = 13	6.40 [4.39; 16.0] *n* = 9	14.06 [9.47; 66.40]^‡^ *n* = 11	
Ferritin, ng/ml	226.5 [80; 324.5] *n* = 6	295 [211; 345] *n* = 7	358 [277; 764] *n* = 7	
Creatinine, *μ*M	90.5 [78; 101.8]	95.5 [86.3; 134]	98 [85.1; 122.5]	
GSH, *μ*M	1.81 [1.04; 2.34]	1.15 [0.86; 1.76]	1.22 [0.76; 1.42]^‡^	
Hcy, *μ*M	7.4 [5.9; 9.3]	8.3 [7.0; 10.5]	9.1 [6.5; 12.8]	
SAM, nM	59 [48; 72]	57 [51; 84]	84 [64, 115]^‡^	
SAH, nM	14.4 [4.4; 19.9]	10.2 [8.0; 18.3]	14.5 [12.2; 24.7]	
SAM/SAH	3.6 [2.7; 5.4]	7.2 [4.2; 9.1]^‡^	5.5 [3.3; 9.3]	
SAM/GSH, nM/*μ*M	32 [23; 52]	57 [36; 131]^‡^	60 [42; 285]^‡^	0.017

^‡^ *p* < 0.05 compared with “CT0,1” group. ^£^*p* < 0.05 compared with “CT2” group. ALT: alanine aminotransferase; AST: aspartate aminotransferase; CRB: C-reactive protein; CT: computed tomography; ESR: the erythrocyte sedimentation rate; GSH: glutathione; HCT: hematocrit; Hcy: homocysteine; HGB: hemoglobin; IL-6: interleukin-6; LI: leukocyte index; MCH: mean erythrocyte hemoglobin; MCHC: mean corpuscular hemoglobin concentration; MCV: mean erythrocyte volume; PLT: platelets; RBC: red blood cells; SAH: S-adenosylhomocysteine; SAM: S-adenosylmethionine; SpO_2_: oxygenation of blood; WBC: white blood cells.

**Table 3:** Association of SAM and SAM-related indicators with the degree of lung injury of patients with coronavirus infection upon admission.

Indicator	*N* ^CT3,4^	*N* ^CT0-2^	RR	*p*	OR	95% CI
SAM > 80 nM	9 of 14	9 of 42	3.0	0.0029	6.6	1.8-24.7
	*N* ^CT2-4^	*N* ^CT0,1^				
SAM/GSH > 60 nM/*μ*M	15 of 30	5 of 26	2.6	0.017	4.2	1.25-14.1
SAM/SAH > 6.0	20 of 30	5 of 26	3.47	0.0004	8.4	2.4-28.9

CT: computed tomography; GSH: glutathione; SAH: S-adenosylhomocysteine; SAM: S-adenosylmethionine.

## Data Availability

The anonymized data are available: https://figshare.com/articles/dataset/SAM_SAH_Covid_patients_data_xls/16539591.

## Abbreviations

2′-OMTase: 2′-O-methyltransferase
Ado: Adenosine
ALT: Alanine aminotransferase
AST: Aspartate aminotransferase
CRB: C-reactive protein
COVID-19: Coronavirus disease 2019
CT: Computed tomography
ED: Endothelial dysfunction
ESR: The erythrocyte sedimentation rate
GGL: Gamma-glutamylcysteine ligase
GGT: Gamma-glutamyl transferase
GSH: Glutathione
HCT: Hematocrit
Hcy: Homocysteine
HGB: Hemoglobin
IL-6: Interleukin-6
LI: Leukocyte index
LPS: Lipopolysaccharide
MAPK: Mitogen-activated protein kinases
MCH: Mean erythrocyte hemoglobin
MCHC: Mean corpuscular hemoglobin concentration
MCV: Mean erythrocyte volume
N7-Mtase: (Guanine-N7)-methyltransferase
NETs: Neutrophil extracellular trap
Nsp: Nonstructural proteins
OR: Odds ratio
PLT: Platelets
RBC: Red blood cells
RR: Relative risk
SAH: S-adenosylhomocysteine
SAHH: SAH hydrolase
SAM: S-adenosylmethionine
SARS-CoV-2: Acute respiratory syndrome coronavirus-2
WBC: White blood cells.
